# Continuous Glucose Monitoring Sensor Glucose Levels and Insulin Pump Infusion Set Wear-Time During Treatment with Fast-Acting Insulin Aspart: A Post Hoc Analysis of Onset 5

**DOI:** 10.1089/dia.2021.0199

**Published:** 2022-01-05

**Authors:** Anders Gorst-Rasmussen, Jeppe Sturis, Magnus Ekelund

**Affiliations:** ^1^ Novo Nordisk A/S, Søborg, Denmark.; ^2^ Novo Nordisk A/S, Måløv, Denmark.

**Keywords:** Continuous glucose monitoring, Continuous subcutaneous insulin infusion, Faster aspart, Glycemic control, HbA1c, Infusion set, Insulin pump, Postprandial blood glucose, Prandial insulin, Type 1 diabetes

## Abstract

***Background:*** In the onset 5 trial, fast-acting insulin aspart (faster aspart) was noninferior to insulin aspart (IAsp) for change from baseline glycated hemoglobin at 16 weeks, when used in continuous subcutaneous insulin infusion by participants with type 1 diabetes. The aim of this post hoc analysis was to investigate whether infusion set wear-time was associated with changes in sensor glucose, measured using continuous glucose monitoring (CGM).

***Materials and Methods:*** This was a post hoc analysis of onset 5 data. Mean infusion set wear-time and duration of CGM-wearing period were assessed. Mean CGM sensor glucose 24 h before and 24 h after were used to calculate the before–after difference (CGM sensor glucose drift).

***Results:*** Mean infusion set wear-time was 2.9 and 3.0 days in the faster aspart and IAsp arms, respectively. At 16 weeks, the average duration of the CGM wearing period was 13.7 and 13.8 days, respectively. Mean CGM sensor glucose before versus after an infusion set change, at week 16, was 10.14 versus 9.39 mmol/L with faster aspart and 9.48 versus 9.47 mmol/L with IAsp. The estimated treatment difference in CGM sensor glucose drift at 16 weeks for faster aspart versus IAsp was +0.72 mmol/L (95% confidence interval: 0.48–0.96, *P* < 0.001).

***Conclusions:*** Mean infusion set wear-time and duration of CGM-wearing period were similar for faster aspart and IAsp. A significantly greater upward drift in CGM sensor glucose values measured during an infusion set wearing period was observed with faster aspart versus IAsp.

***Clinical trial registration:*** NCT02825251.

## Background

The use of continuous subcutaneous insulin infusion (CSII) with an insulin pump may be considered as a treatment option in type 1 diabetes (T1D), regardless of the individual's age.^1^ Meta-analyses of randomized controlled trials have shown an association between CSII treatment and improved glycemic control with a lower risk of severe hypoglycemia, compared with multiple daily injections (MDI).^2^ However, reaching treatment goals in people with T1D remains challenging.^3^ The potential to improve attainment of targets may lie in the development of more advanced CSII treatment that uses automated, closed-loop medical devices and real-time data from continuous glucose monitoring (CGM) systems to inform mathematical algorithms that control insulin and/or glucagon delivery. However, insulin kinetics remains a major limitation of present and near-future insulin delivery systems.^4^

Fast-acting insulin aspart (faster aspart) is a formulation of insulin aspart (IAsp) with the additional excipients l-arginine and niacinamide.^5,6^ In a pooled analysis of phase 1 data, faster aspart demonstrated an earlier onset of appearance and a greater early insulin exposure compared with IAsp in adults with T1D when administered subcutaneously.^5^ Furthermore, a greater glucose-lowering effect was reported in participants with T1D using CSII.^7,8^ In the phase 3 onset 5 trial, the estimated treatment difference (ETD) for change from baseline in glycated hemoglobin (HbA1c) 16 weeks after randomization was 0.09% (95% confidence interval [CI]: 0.01–0.17, *P* < 0.001), confirming noninferiority of faster aspart versus IAsp. However, although noninferiority was observed, the clinically minor treatment difference was statistically significantly in favor of IAsp versus faster aspart. In addition, faster aspart was significantly superior to IAsp for controlling postprandial glucose (PPG), as assessed by 1-h PPG increment after a meal test.^6^ CGM data from the study reported broadly similar CGM sensor glucose values (i.e., interstitial glucose) between the faster aspart group and the IAsp group at 16 weeks, however, there was a trend toward higher median values during the night time compared with baseline in the faster aspart group.^6^ A previous trial demonstrated a statistically significant difference in favor of faster aspart for change in PPG in people with T1D using MDI.^9^ Furthermore, improvements in 30-min, 1-h, and 2-h PPG increments was supported by CGM glucose values in the onset 5 trial.^6^ Therefore, the lack of translation of improved PPG into an improvement in HbA1c with faster aspart versus IAsp in the onset 5 trial warrants further investigation.

The pharmacokinetics (PK)/pharmacodynamics (PD) of insulin delivered by pump varies somewhat during the wear-time of an infusion set.^10,11^ Furthermore, infusion set wear-time has been shown to be positively correlated with increases in blood glucose (BG) levels.^12^ The aim of the present post hoc analysis was to explore the association between infusion set wear-time and changes in CGM sensor glucose.

## Materials and Methods

### Study design

The trial design of onset 5 has been described previously.^6^ In brief, onset 5 was a double-blind randomized multicenter parallel-group treat-to-target trial in adults with T1D administered with faster aspart versus IAsp through CSII (NCT02825251). Eligible participants were adults (≥18 years) with T1D, who had an HbA1c of 7.0%–9.0% and had been using the same insulin pump with a rapid-acting insulin analog for ≥6 months.

During the 4-week run-in period, participants remained on their pretrial insulin, and basal pump rates and bolus dose calculator settings were only adjusted for safety reasons. After the run-in period, participants were randomized to treatment with either faster aspart or IAsp delivered through CSII for a 16-week treatment period. During the treatment period, basal rates were adjusted to keep BG in a stable range (within 2 mmol/L [35 mg/dL]). Mealtime insulin was titrated based on carbohydrate counting according to usual practice. Basal rates, insulin:carbohydrate ratios, insulin sensitivity factors and active insulin were adjusted at the investigator's discretion.

The onset 5 trial was conducted according to the Declaration of Helsinki Amended 2013 and International Conference on Harmonization Good Clinical Practice (1996). Before trial initiation, the protocol, the consent form, and the subject information sheet were reviewed and approved according to local regulations by appropriate health authorities, and by Institutional Review Boards. A full list of the research ethics boards/institutional review boards, with their reference numbers, is provided in Supplementary Table S1 in the Supplementary Data. All patients provided written informed consent.

### CGM devices

At most, 50% of participants were allowed to use their own (unblinded) CGM device during the trial. Randomization was stratified according to use of own unblinded CGM device. All participants received a blinded CGM device (Dexcom G4) that was worn before randomization, before the 8th week after randomization, and before the 16th week after randomization. Participants were instructed to use their sponsor-administered BG-meter to do daily calibration of the blinded CGM based on at least two self-measured BG measurements.

### Infusion set changes

Participants were provided with infusion sets during site visits and were instructed to routinely change their infusion set in intervals not exceeding 3 days (2 days for Sure T [Easy Set]) and as such, timing of infusion set change was selected by each participant. Infusion sets and reservoirs should have been changed at the same time, and participants were instructed to use the same infusion region (preferably the abdominal wall) throughout the trial and to rotate the infusion site within the region. All infusion set changes and timings were recorded in participants' diaries.

### Post hoc analysis endpoints

**FIG. 1.**
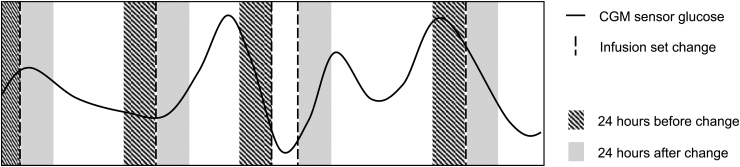
Illustration of how the mean CGM sensor glucose before and after an infusion change were calculated for each participant, when restricting to wear-times to ≥24 h. Mean CGM sensor glucose 24 h before an infusion set change is calculated using all of the CGM sensor glucose values in the hashed areas. Mean CGM sensor glucose 24 h after an infusion set change is calculated using all of the CGM sensor glucose values in the hashed solid gray areas. Only CGM sensor glucose data from periods in which the infusion set was worn for ≥24 h were included in the analysis. CGM, continuous glucose monitoring.

Mean wear-time was the mean length of time participants wore an infusion set. Participants were requested to record in a diary the date and time of infusion set change, and whether it was a routine change. The mean duration of the CGM wearing period was the number of days between the first and last observed reading from the CGM device. The mean of all available CGM sensor glucose values from devices in the 24-h period before, and the 24-h period after, an infusion set change was calculated for each participant. Only CGM sensor glucose data from periods in which the infusion set was worn for ≥24 h were included in the analysis. Figure 1 illustrates, schematically, how CGM data were used in this analysis.

The within-participant difference in mean CGM sensor glucose over all 24-h periods before minus 24-h periods after an infusion set change was calculated. This will be referred to as the “CGM sensor glucose drift,” as it quantifies the average change occurring over a wear-time typical for that participant (of duration ≥24 h). Considering a complete 24-h period before and after an infusion set change accounted for the impact of diurnal variation in CGM glucose.

Following a similar approach as for CGM sensor glucose drift, the total number of hypoglycemic episodes and bolus dose in the 24-h period before, and the 24-h period after, an infusion set change was calculated for each participant, restricting data to wearing periods of ≥24 h. The rate of hypoglycemia was calculated as number of events per year at risk.

### Statistical analysis

In alignment with the primary analyses of onset 5,^6^ the current post hoc analyses were based on all randomized participants for the entire duration of the trial, and thus included data collected after premature discontinuation of trial product (intention-to-treat).

An analysis of covariance (ANCOVA) model was applied to the CGM sensor glucose drift endpoint at 16 weeks after randomization, adjusting for the effect of treatment, stratum, and baseline CGM sensor glucose drift. ETDs in CGM sensor glucose drift (faster aspart–IAsp) were reported with 95% CI and two-sided *P*-values for the test of no difference. CGM sensor glucose drift was also analyzed by subgroups defined by needle length, tubing length, infusion set type, pump type, and use of own CGM. These subgroup analyses were performed using a similar ANCOVA model but including the interaction between the subgroup variable and treatment as a fixed factor, and reporting interaction *P*-values for the test of no heterogeneity between subgroups.

## Results

In the onset 5 trial, 472 participants were randomized to CSII treatment with faster aspart (*n* = 236; mean age: 43.3 years) or IAsp (*n* = 236; mean age: 43.6 years).^6^ Overall, ∼25% of participants in each treatment arm used their own CGM device. Participants in the faster aspart and IAsp treatment arm had a mean body mass index (BMI) of 26.2 and 26.5 kg/m^2^, respectively, and mean HbA1c was 7.49% in both treatment arms. The mean duration of diabetes was 25.0 and 23.3 years in the faster aspart and IAsp arms, respectively. Previous IAsp use was reported in 53.4% and 60.2% participants in the faster aspart and IAsp arms, respectively. Pump model and infusion set at screening are shown in Table 1.^6^

**Table 1.** Pump Model and Infusion Set at Screening

	Faster aspart	IAsp	Total
*N*	236	236	472
Pump model at screening, %			
Paradigm Veo^a^	132 (55.9)	119 (50.4)	251 (53.2)
Minimed 530G^a^	47 (19.9)	49 (20.8)	96 (20.3)
Paradigm	35 (14.8)	35 (14.8)	70 (14.8)
Paradigm Revel	22 (9.3)	33 (14.0)	55 (11.7)
Infusion set first dispensed^b^, %			
Quick-set	154 (65.3)	170 (72.0)	324 (68.6)
Silhouette	41 (17.4)	35 (14.8)	76 (16.1)
Mio	24 (10.2)	19 (8.1)	43 (9.1)
Sure-T (Easy set)	17 (7.2)	12 (5.1)	29 (6.1)

*N*, number of participants.

The recommended frequency for changing each infusion set was every 3 days for the Quick-set, Silhouette, and Mio, and every 2 days for the Sure-T, as per the manufacturers' instructions.

^a^ Low glucose suspend feature not allowed as per protocol.

^b^ Participants were free to change infusion sets during the trial.

© John Wiley and Sons 2019, reproduced with permission from Klonoff et al.^6^

Faster aspart, fast-acting insulin aspart; IAsp, insulin aspart.

### Mean infusion set wear-time

**FIG. 2.**
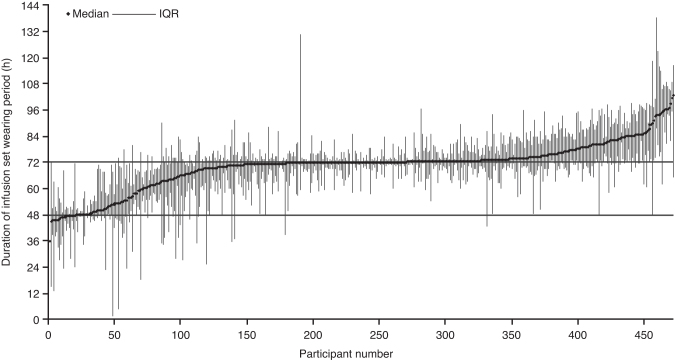
Distribution of infusion set wear-times. IQR, interquartile range.

The distribution of infusion set wear-times by participants indicated a clustering around a median wear-time of 72 h, and showed that many participants had wear-times >48 h (Fig. 2). When restricting measurement to infusion set wear-times of a duration ≥24 h, mean wear-time was 2.9 days in the faster aspart arm and 3.0 days in the IAsp arm. Similar mean wear-times were observed when considering the different infusion sets (faster aspart: 3.0–3.1 days; IAsp: 2.9–3.1 days), except for Sure-T (Easy set), for which the mean wear-time was 2.2 days in both treatment arms.

### Mean duration of CGM wearing period

**FIG. 3.**
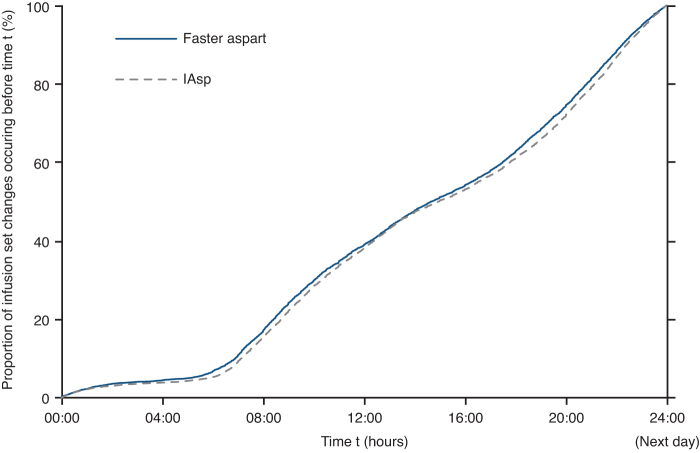
Distribution of timing of infusion set change by treatment arm. Faster aspart, fast-acting insulin aspart; IAsp, insulin aspart.

At 16 weeks, the mean duration of the CGM wearing period was 13.7 days in the faster aspart group and 13.8 days in the IAsp group. Few infusion set changes occurred during the night (midnight to 06:00), whereas changes occurred at an approximately uniform rate throughout the daytime (Fig. 3).

### Mean CGM sensor glucose

**FIG. 4.**
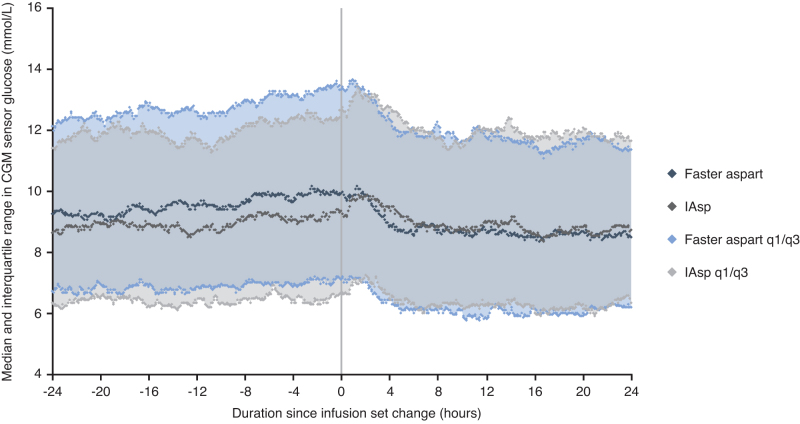
Median and interquartile range of CGM sensor glucose values by treatment before and after infusion set change at 16 weeks, restricted to wear-times ≥24 h. CGM, continuous glucose monitoring; faster aspart, fast-acting insulin aspart; IAsp, insulin aspart; IQR, interquartile range.

Descriptive summary statistics for CGM sensor glucose values by week, before and after an infusion set change, are presented in Table 2. The Week 16 results were consistent with Figure 4, indicating a higher mean CGM sensor glucose before versus after an infusion set change with faster aspart (10.14 vs. 9.39 mmol/L), but not with IAsp (9.48 vs. 9.47 mmol/L) (Table 2). Similar results were seen at week 8, but not at baseline, where participants in both arms were receiving their prerandomization insulin.

**Table 2.** Mean CGM Sensor Glucose Values 24 h Before and After Infusion Set Change by Treatment Arm, Restricted to Wear-Times ≥24 h

	Mean CGM sensor glucose (mmol/L)	
	Faster aspart	IAsp
Week 0, *N*	235	234
−24 to 0 h	9.59 (1.50)	9.67 (1.55)
0 to 24 h	9.43 (1.52)	9.31 (1.39)
Week 8, *N*	231	229
−24 to 0 h	10.08 (1.54)	9.54 (1.47)
0 to 24 h	9.52 (1.45)	9.39 (1.40)
Week 16, *N*	228	224
−24 to 0 h	10.14 (1.60)	9.48 (1.43)
0 to 24 h	9.39 (1.64)	9.47 (1.38)

Values are mean (standard deviation), *N* indicates the number of participants contributing to the summary statistics.

CGM, continuous glucose monitoring; faster aspart, fast-acting insulin aspart; IAsp, insulin aspart.

The week 16 median and interquartile range of CGM sensor glucose values 24 h before and after an infusion set change are presented in Figure 4. In the faster aspart arm, CGM sensor glucose values were higher before versus after an infusion set change, whereas in the IAsp arm values remained relatively unchanged after an infusion set change. Data were aligned to time of infusion set change; therefore, the median and quartiles were based on CGM sensor glucose values at different clock times.

### CGM sensor glucose drift

**FIG. 5.**
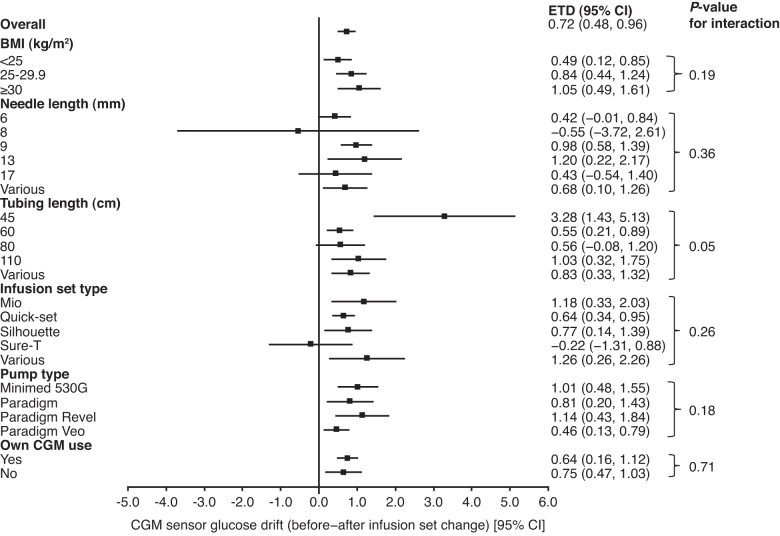
Statistical analysis of CGM sensor glucose drift over a typical wearing period at 16 weeks, restricted to wear-times ≥24 h. For needle length, tubing length, and infusion set types participants who were dispensed a given type throughout the trial were assigned to that category; participants dispensed multiple types are assigned to the “various” category. Treatment differences were estimated using ANCOVA. The CGM sensor glucose drift is the within-participant difference in mean CGM sensor glucose over all 24-h periods before versus 24-h periods after an infusion set change. ANCOVA, analysis of covariance; BMI, body mass index; CGM, continuous glucose monitoring; CI, confidence interval; ETD, estimated treatment difference.

Statistical analysis of the CGM sensor glucose drift over a typical wearing period at 16 weeks is shown in Figure 5. Overall, the ETD at week 16 was +0.72 mmol/L (95% CI: 0.48–0.96, *P* < 0.001), indicating a significantly greater CGM sensor glucose drift during an infusion set wearing period with faster aspart compared with IAsp. These results were consistent across subgroup analyses by BMI groups, needle length, tubing length, infusion set type, pump type, and whether own CGM was used (Fig. 5). There was no evidence of heterogeneity within subgroups, as indicated by nonsignificant interaction *P*-values, except for tubing length (*P* = 0.05) where a larger treatment difference was seen for the shortest tubing length; however, only eight participants contributed data in this subgroup.

### Insulin dose

During infusion set wearing periods of duration ≥24 h, the bolus insulin dose delivered in the 24 h before versus after an infusion set change was similar for faster aspart (25.9 vs. 25.9 U) and IAsp (24.8 vs. 25.9 U). The mean total daily insulin dose at 16 weeks was similar between faster aspart and IAsp (49.72 vs. 49.12 U). Although individualized adjustments of basal rate settings occurred during the trial, no systematic differences between treatment arms were observed.

### Hypoglycemia

The rate of BG-confirmed or severe hypoglycemic episodes was numerically higher after versus before an infusion set change with both faster aspart and IAsp; however, the difference after versus before was numerically greater for faster aspart (46.5/participant-year of exposure [PYE] vs. 27.3/PYE) than for IAsp (39.1/PYE vs. 32.0/PYE). The rate of severe hypoglycemic episodes was low in both arms both before (faster aspart: 0.13/PYE; IAsp: 0.10/PYE) and after infusion set change (faster aspart: 0.25/PYE; IAsp: 0.06/PYE).

## Discussion

In this post hoc analysis of onset 5, there was a statistically significant upward drift in the CGM sensor glucose values during a typical infusion set wearing period with faster aspart compared with IAsp. Overall, the treatment difference in the CGM sensor glucose drift was +0.72 mmol/L from the first to the last day of an infusion set wearing period, with the mean infusion set wear-time of ∼3 days in each treatment arm. Similar results were observed across subgroups defined by needle length, tubing length, infusion set type, pump type, and use of own CGM. Timing of hypoglycemic episodes was consistent with the finding of an upward drift with faster aspart, with the rate of BG-confirmed or severe hypoglycemic episodes attenuated the last day before, versus the first day after, an infusion set change.

The estimated difference in CGM sensor glucose drift between treatments of +0.72 mmol/L reflects a population average. For the individual participant, the drift is less likely to be noticeable in daily life, considering the benefits that faster aspart provides in relation to PPG control.^6^ There was no evidence that more bolus insulin (and, therefore, correction doses) was given on the last day of an infusion set wearing period with faster aspart compared with IAsp. Nevertheless, the increased CGM sensor glucose drift seen with faster aspart compared with IAsp may drive the minor differences in glycemic control observed in onset 5 where the ETD for change from baseline in HbA1c was 0.09%, favoring IAsp. Indeed, at 16 weeks the mean CGM sensor glucose during the first 24 h after an infusion set change was similar between faster aspart and IAsp (9.39 vs. 9.47 mmol/L). For comparison, the overall mean CGM sensor glucose among all randomized participants at week 16 was 9.66 mmol/L for faster aspart versus 9.41 mmol/L for IAsp.

Treatment-emergent adverse events in the two treatment arms have already been reported.^6^ Overall, the frequency of infusion-site reactions that were considered possibly or probably related to trial product was numerically higher in the faster aspart arm, occurring among 5.5% of participants (0.29 events/PYE) versus 3.8% of participants (0.18 events/PYE) in the IAsp arm.^6^

Comparing the effectiveness of bolus insulins used in pumps can be challenging, as differences in effectiveness may also be due to differences in pump device type and settings. In the onset 5 trial, participants were required to turn off the automatic feature of the pump (low glucose suspend mode) and to wear a blinded CGM device, to ensure that the comparison between faster aspart and IAsp was not impacted by device-driven compensation.^6^ However, it is inherently difficult to successfully separate the effects of drug and device. Although there are no likely compensatory settings in conventional CSII that could mitigate drift over the duration of an infusion set wearing period, CSII with automated insulin delivery may be able to address issues related to CGM sensor glucose drift. For example, a randomized trial of adults with T1D was conducted to investigate the safety of, and glucose control by, faster aspart in the insulin-only configuration of the iLet^®^ bionic pancreas (iLet; Beta Bionics, Inc., Concord, MA), fully automated insulin delivery system.^13^ The results suggest that glucose control is even further improved when the time to maximal serum drug concentration (*t*_max_) setting is adapted to the pharmacological profile of faster aspart. There were no safety concerns with faster aspart using the iLet with nondefault *t*_max_ settings, and improvements were observed in mean sensor glucose values without increases in low sensor glucose.^13^

The mechanism of the CGM sensor glucose drift is not clear. The PK of insulin in a pump changes over the lifetime of the infusion set,^10,11^ and subcutaneous adipose tissue blood flow at the site of infusion varies during the catheter wear-time.^10^ Faster aspart differs from IAsp in that it contains two additional excipients: niacinamide and l-arginine.^14^ Niacinamide has been demonstrated to induce vasorelaxation of preconstricted small arteries in vitro and to induce vasodilation in vivo after bolus injection in pigs,^14^ but it is not known if the degree of vasodilation and effect on IAsp absorption is constant over time in a pump setting, or how it potentially interacts with the variation over time described by Clausen et al.^10^

It is, therefore, conceivable that a differential development of the PK profile occurs with faster aspart compared with IAsp, making it more difficult to maintain constant glycemic control when the infusion set has been in place for several days. Testing this hypothesis would require assessment of the PK and PD over time using the same infusion set and catheter. Such a trial would also allow investigation of the preferred frequency of infusion set changes, to mitigate CGM sensor glucose drift with faster aspart. This may allow clinicians to recommend to patients using faster aspart the time interval at which they should change their infusion set. For such a trial, infusion set changes would be imposed at specific time intervals and the CGM sensor glucose drift assessed and compared between treatments.

The rate of severe or BG-confirmed hypoglycemia was numerically higher after an infusion set change versus before, with both faster aspart and IAsp. It is worth noting that data for the reported hypoglycemic events and data for the CGM sensor glucose were from different sources and, therefore, these results are internally validated because both these sets of data support each other.

Strengths of the present analysis include the randomized controlled design of the original study, the relatively long duration of CGM wearing periods, and the comprehensive diary-based time recording of infusion set changes. Limitations include the self-selected duration of infusion set wearing periods, which complicate the evaluation of the association between wear-time and CGM glucose values, as participants contribute different wear-times. This was mitigated by deriving a measure of within-participant drift in CGM sensor glucose during a wearing period typical for that participant; this was unambiguously calculated for all participants. Another limitation was that glycemia was measured through CGM, which is dependent on correct calibration.

## Conclusions

In conclusion, this post hoc analysis of the onset 5 trial showed a drift upward in CGM sensor glucose values during a typical infusion set wearing period with faster aspart versus IAsp. The reasons for this remain unknown, but it was hypothesized that CSII with more automated insulin delivery could compensate for this drift upward, and thereby may help leverage the improved time-action profile of faster aspart versus IAsp.

## Compliance with Ethics Guidelines

All procedures followed in the onset 5 study were in accordance with the ethical standards of the responsible committee on human experimentation (institutional and national) and with the Declaration of Helsinki 1975, as revised in 2013. A full list of the research ethics boards/institutional review boards, with their reference numbers, is provided in Supplementary Table S1 in the Supplementary Data. Informed consent was obtained from all participants for being included in the trial.

## Supplementary Material

Supplemental data
