# Supplementary material for: Continuous Glucose Monitoring Sensor Glucose Levels and Insulin Pump Infusion Set Wear-Time During Treatment with Fast-Acting Insulin Aspart: A Post Hoc Analysis of Onset 5
Source: Diabetes Technol Ther. 2022 Jan 5;24(1):10–7. doi: 10.1089/dia.2021.0199 (PMC8783624; doi:10.1089/dia.2021.0199)
Supplement: Supplemental data [file Supp_Data.pdf]

CGM sensor glucose levels and insulin pump infusion set wear-time during treatment with fast-acting insulin aspart: a *post hoc* analysis of onset 5

Anders **Gorst-Rasmussen**, PhD, Jeppe **Sturis**, PhD, Magnus **Ekelund**, MD, PhD

Supplementary Information

The trial protocol and any applicable protocol amendments were submitted as shown below for review, according to local requirements, by an independent ethics committee (IEC), i.e. a review panel responsible for ensuring the protection of the rights, safety, and well-being of human subjects involved in a clinical investigation. The IEC was adequately constituted to provide assurance of that protection according to the requirements of ICH GCP.

Table S1: List of Independent Ethics Committees/Institutional Review Boards

Site no.	Investigator name<br>Address	IEC Name<br>Address<br>Chairperson
101	Professor Christophe De Block<br>UZ Antwerpen<br>Department of endocrinology, metabolic diseases and nutrition pathology<br>Wilrijkstraat 10<br>B-2650 Edegem	Ethics Committee UZA<br>Wilrijkstraat 10<br>B-2650 Edegem<br>Prof Dr Patrick Cras
102	Dr Pascale Abrams<br>GZA Sint-Augustinus<br>Department of Endocrinology<br>Oosterveldlaan 24<br>B-2610 Wilrijk	Commissie Medische Ethiek GZA<br>Toetsingskamer<br>GZA Ziekenhuizen<br>Oosterveldlaan 24<br>B-2610 Wilrijk<br>Prof Dr Bart Van Den Eynden
103	Prof Dr Bart Keymeulen<br>UZ Brussel<br>Diabeteskliniek<br>Laarbeeklaan 101<br>B-1090 Brussel	Commissie Medische Ethiek<br>UZ Brussel<br>Grote Prefab. Niv. 1<br>Laarbeeklaan 101<br>B-1090 Brussel<br>Prof Dr Em André Van Steirteghem

104	Prof Dr Chantal Mathieu<br>UZ Leuven<br>Campus Gasthuisberg<br>Department of endocrinology<br>Herestraat 49<br>B-3000 Leuven	Commissie Medische Ethiek van Universitaire Ziekenhuizen<br>K.U. Leuven<br>Campus Gasthuisberg E330<br>Herestraat 49<br>B-3000 Leuven<br>Prof Dr Ivo De Wever
105	Dr Chris Vercammen<br>AZ Imelda<br>Department of Endocrinology<br>Imeldalaan 9<br>B-2820 Bonheiden	Commissie Medische Ethiek<br>Imelda vzw<br>Imeldalaan 9<br>B-2820 Bonheiden<br>Dr Stijn Gysenbergs
106	Dr Eric Weber<br>CSL-Vivalia<br>Department of Endocrinology-Diabetology<br>Rue des Déportés 137<br>B-6700 Arlon	Ethics Committee<br>Cliniques du Sud Luxembourg<br>Rues des Déportés 137<br>B-6700 Arlon<br>Dr Philippe Gobert
107	Dr Peter Coremans<br>AZ Nikolaas<br>Department of Endocrinology<br>Moerlandstraat 1<br>B-9100 Sint-Niklaas	MMA<br>Kwaliteitszorg/toetsingscommissie<br>AZ Nikolaas vzw<br>Moerlandstraat 1<br>B-9100 Sint-Niklaas<br>Dr Gino De la Meilleure
201	Dr. Hani G. Alasaad<br>LMC Diabetes and Endocrinology (Barrie)<br>5 Quarry Ridge Road, Suite 306<br>Barrie, ON L4M 7G1	Institutional Review Board Services (IRBS)<br>372 Hollandview Trail, Suite 300 Aurora, ON L4G 0A5<br>Marianne Vanderwel<br><br>Anna Carnavale: Senior Coordinator – Client Services
202	CRCHUM (Rm R08-420)<br>900, rue St-Denis<br>Montréal, QC H2X 0A9	Comite d'ethique de la recherche du CHUM CéR / Comite d'ethique de la recherche du CHUM CéS<br>900 Rue St-Denis, 3e étage, Montreal, QC, H2X OA9<br><br>Chairperson: Camille Assemat,Vice-présidente, Comite d'ethique de la recherche du CHUM<br><br>Chairperson: Dr. Michel Boivin, Président du CéS du CHUM

203	Dr. Ronald M. Goldenberg<br>LMC Diabetes and Endocrinology (Thornhill)<br>531 Atkinson Avenue, Suite 17<br>Thornhill, ON L4J 8L7	Institutional Review Board Services (IRBS)<br>372 Hollandview Trail, Suite 300 Aurora, ON L4G 0A5<br>Marianne Vanderwel<br><br>Anna Carnavale: Senior Coordinator – Client Services
204	Dr. Loren David Grossman<br>LMC Diabetes and Endocrinology (Bayview)<br>1929 Bayview Avenue, Suite 107<br>Toronto, ON M4G 3E8	Institutional Review Board Services (IRBS)<br>372 Hollandview Trail, Suite 300 Aurora, ON L4G 0A5<br>Marianne Vanderwel<br><br>Anna Carnavale: Senior Coordinator – Client Services
205	Dr. Irene Mary Hramiak<br>St. Josephs Health Care<br>268 Grosvenor Street<br>Room B 5- 656<br>London, ON N6A 4V2	Western University Health Science Research Ethics Board Western University, Research, Support Services Building, Suite 5150 London, ON N6G 1G9<br><br>Chairperson: Dr. Marcelo Kremenchytzky, HSREB; Vice Chair Nicole Kaniki, Ethics Officer
206	Dr. Peter A Senior<br>2-004 Li Ka Shing Centre for Health Research Innovation<br>87th Ave 112th Street<br>Edmonton, AB T6G 2E1	University of Alberta Research Ethics Board<br>8625 112 Street – Campus Tower, Suite 308<br>Edmonton, AB T6G 2E1<br>Dr. Shane Kimber, Chair
207	Dr. S. John Weisnagel (Stanley John)<br>CHU de Québec-Université Laval<br>2705 Boul. Laurier TR 27<br>Québec, QC G1V 4G2	CHU de Quebec Université Laval 10, Rue de l"Espinay local A0-124 Quebec, QC G1L 3L5<br><br>Chariperson: Catherine Vayssier, Présidente du Comité de convenance, CHU de Quebec-Université Laval<br><br>Comite d'ethique de la recherche du CHUM CéR / Comite d'ethique de la recherche du CHUM CéS<br>900 Rue St-Denis, 3e étage, Montreal, QC, H2X OA9<br><br>Chairperson: Camille Assemat,Vice-présidente, Comite d'ethique de la recherche du CHUM<br><br>Chairperson: Dr. Michel Boivin, Président du CéS du CHUM

208	Dr. David Yaw Twum-Barima LMC<br>Diabetes and Endocrinology<br>(Oakville)<br>3075 Hospital Gate, Suite 301<br>Oakville, ON L6M 1M1	Institutional Review Board Services<br>(IRBS)<br>372 Hollandview Trail, Suite 300 Aurora, ON<br>L4G 0A5<br><br>Chairperson: Marianne Vanderwel<br>Chair<br><br>Anna Carnavale Senior Coordinator –<br>Client Services
400	Dr. med. Thomas Behnke<br>Zentrum für Klinische Studien Neuwied<br>Langendorfer Str. 82a<br>56564 Neuwied	Ethikkommission der Ärztekammer<br>Westfalen-Lippe und der<br>Westfälischen Wilhelms-Universität<br>Gartenstra13e 210-214<br>48147 Münster<br>Prof. Dr. med. Wolfgang E. Berdel
401	Dr. med. Hans-Peter Kempe<br>Ludwigsplatz 9<br>67059 Ludwigshafen	Ethikkommission der Ärztekammer<br>Westfalen-Lippe und der<br>Westfälischen Wilhelms-Universität<br>Gartenstra13e 210-214<br>48147 Münster<br>Prof. Dr. med. Wolfgang E. Berdel
402	Dr. med. Ludger Rose<br>Institut für Diabetesforschung GmbH<br>Hohenzollernring 70<br>48145 Münster	Ethikkommission der Ärztekammer<br>Westfalen-Lippe und der Westfälischen<br>Wilhelms-Universität Gartenstra13e 210-<br>214<br>48147 Münster<br>Prof. Dr. med. Wolfgang E. Berdel
403	Dr. med. Armin Sammler<br>Zum Grühlingsstollen 3<br>66299 Friedrchsthal	Ethikkommission der Ärztekammer<br>Westfalen-Lippe und der<br>Westfälischen Wilhelms-Universität<br>Gartenstra13e 210-214<br>48147 Münster<br>Prof. Dr. med. Wolfgang E. Berdel
404	Dr. med. Simon Vidal<br>Diabetespraxis Bad Mergentheim<br>Theodor-Klotzbücher-Str.12<br>97980 Bad Mergentheim	Ethikkommission der Ärztekammer<br>Westfalen-Lippe und der<br>Westfälischen Wilhelms-Universität<br>Gartenstra13e 210-214<br>48147 Münster<br>Prof. Dr. med. Wolfgang E. Berdel
405	Dr. med. Ulrich Wendisch<br>Beselerstr. 2a<br>22607 Hamburg	Ethikkommission der Ärztekammer<br>Westfalen-Lippe und der<br>Westfälischen Wilhelms-Universität<br>Gartenstra13e 210-214<br>48147 Münster<br>Prof. Dr. med. Wolfgang E. Berdel

406	Dr. med. Veronika Wenzl-Bauer<br>Bahnhofstr. 1a<br>66780 Rehlingen Siersburg	Ethikkommission der Ärztekammer Westfalen-Lippe und der Westfälischen Wilhelms-Universität<br>Gartenstra13e 210-214<br>48147 Münster<br>Prof. Dr. med. Wolfgang E. Berdel
407	Dr. med. Helga Zeller-Stefan<br>Diabetes Zentrum<br>Eleonorastr. 42<br>45136 Essen	Ethikkommission der Ärztekammer Westfalen-Lippe und der Westfälischen Wilhelms-Universität<br>Gartenstra13e 210-214<br>48147 Münster<br>Prof. Dr. med. Wolfgang E. Berdel
408	Dr. med. Stephan Arndt<br>diabendo Praxiszentrum<br>Doberaner Str. 115<br>18057 Rostock	Ethikkommission der Ärztekammer Westfalen-Lippe und der Westfälischen Wilhelms-Universität<br>Gartenstra13e 210-214<br>48147 Münster<br>Prof. Dr. med. Wolfgang E. Berdel
409	Dr. med. Andreas Staudenmeyer<br>Am Wall Süd 20<br>49808 Lingen	Ethikkommission der Ärztekammer Westfalen-Lippe und der Westfälischen Wilhelms-Universität<br>Gartenstra13e 210-214<br>48147 Münster<br>Prof. Dr. med. Wolfgang E. Berdel
301	Pr Yves REZNIK, MD<br>Centre Hospitalier Universitaire<br>Service d'Endocrinologie Diabétologie<br>Avenue de la Côte de Nacre<br>14033 CAEN Cedex 9<br>FRANCE	Comité de Protection des Personnes Nord-Ouest III<br>Centre Hospitalier Universitaire<br>Niveau 3 - porte 508<br>Avenue de la Côte de Nacre<br>CS 30001<br>14033 CAEN cedex 9<br>Charlotte Gourio
302	Pr Hélène HANAIRE, MD<br>Hôpital de Rangueil<br>Diabétologie - Maladies Métaboliques - Nutrition<br>1, avenue du Professeur Jean Poulhès<br>31059 TOULOUSE cedex 9<br>FRANCE	Comité de Protection des Personnes Nord-Ouest III<br>Centre Hospitalier Universitaire<br>Niveau 3 - porte 508<br>Avenue de la Côte de Nacre<br>CS 30001<br>14033 CAEN cedex 9<br>Charlotte Gourio
303	Dr Sylvaine CLAVEL, MD<br>Hôtel Dieu Le Creusot<br>Service de Diabétologie 26 rue d'Harfleur<br>71200 LE CREUSOT<br>FRANCE	Comité de Protection des Personnes Nord-Ouest III<br>Centre Hospitalier Universitaire<br>Niveau 3 - porte 508<br>Avenue de la Côte de Nacre<br>CS 30001<br>14033 CAEN cedex 9<br>Charlotte Gourio

	Service de Diabétologie 26 rue d'Harfleur<br>71200 LE CREUSOT<br>FRANCE	Centre Hospitalier Universitaire<br>Niveau 3 - porte 508<br>Avenue de la Côte de Nacre<br>CS 30001<br>14033 CAEN cedex 9<br>Charlotte Gourio
304	Dr Didier GOUET, MD<br>Hôpital Saint-Louis Service<br>d'Endocrinologie Rue du Dr Schweitzer<br>17019 LA ROCHELLE FRANCE	Comité de Protection des Personnes<br>Nord-Ouest III<br>Centre Hospitalier Universitaire<br>Niveau 3 - porte 508<br>Avenue de la Côte de Nacre<br>CS 30001<br>14033 CAEN cedex 9
305	Dr Pierre SERUSCLAT, MD<br>Groupe Hospitalier « Les portes du sud »<br>Service d'endocrinologie<br>2, avenue du 11 novembre 1918<br>69200 Vénissieux<br>FRANCE	Comité de Protection des Personnes<br>Nord-Ouest III<br>Centre Hospitalier Universitaire<br>Niveau 3 - porte 508<br>Avenue de la Côte de Nacre<br>CS 30001<br>14033 CAEN cedex 9<br>Charlotte Gourio
306	Pr Bertrand CARIOU, MD<br>Hôpital Nord- Laënnec<br>Service d'Endocrinologie<br>Boulevard Jacques Monod<br>44093 SAINT HERBLAIN<br>FRANCE	Comité de Protection des Personnes<br>Nord-Ouest III<br>Centre Hospitalier Universitaire<br>Niveau 3 - porte 508<br>Avenue de la Côte de Nacre<br>CS 30001<br>14033 CAEN cedex 9<br>Charlotte Gourio
307	Pr Alain PRADIGNAC, MD<br>CHU Hautepierre<br>Service de Médecine Interne,<br>Endocrinologie et nutrition Avenue Molière<br>67098 STRASBOURG Cedex 2<br>FRANCE	Comité de Protection des Personnes<br>Nord-Ouest III<br>Centre Hospitalier Universitaire<br>Niveau 3 - porte 508<br>Avenue de la Côte de Nacre<br>CS 30001<br>14033 CAEN cedex 9<br>Charlotte Gourio
	Pr Eric RENARD, MD<br>AMTIM<br>371 av du Doyen Gaston Giraud<br>34295 MONTPELLIER CEDEX 5<br>Sites satellites:	Comité de Protection des Personnes<br>Nord-Ouest III<br>Centre Hospitalier Universitaire<br>Niveau 3 - porte 508<br>Avenue de la Côte de Nacre

308	Hôpital Lapeyronie – site lié à l'AMTIM<br>Département d'endocrinologie, nutrition et diabète<br>371 av du Doyen Gaston Giraud<br>34295 MONTPELLIER CEDEX 5<br><br>Hôpital St Eloi– site lié à l'AMTIM<br>CIC P-1001<br>80 Av Augustin Fliche<br>34295 MONTPELLIER CEDEX 5	CS 30001<br>14033 CAEN cedex 9<br>Charlotte Gourio
309	Dr Jean François THUAN<br>CHG de Narbonne<br>Service de medecine A<br>Bd Dr LACROIX<br>11108 NARBONNE cedex	Comité de Protection des Personnes Nord-Ouest III<br>Centre Hospitalier Universitaire<br>Niveau 3 - porte 508<br>Avenue de la Côte de Nacre<br>CS 30001<br>14033 CAEN cedex 9<br>Charlotte Gourio
310	Dr Jean-Pierre RIVELINE, MD<br>Hôpital Lariboisière<br>Service de Diabétologie et d'Endocrinologie<br>2 rue Ambroise Paré<br>75010 PARIS	Comité de Protection des Personnes Nord-Ouest III<br>Centre Hospitalier Universitaire<br>Niveau 3 - porte 508<br>Avenue de la Côte de Nacre<br>CS 30001<br>14033 CAEN cedex 9<br>Charlotte Gourio
311	Pr Charles THIVOLET, MD<br>Centre Hospitalier Lyon Sud<br>Pavillon médical<br>165, Chemin du grand Revoyet<br>69495 PIERRE BENITE cedex	Comité de Protection des Personnes Nord-Ouest III<br>Centre Hospitalier Universitaire<br>Niveau 3 - porte 508<br>Avenue de la Côte de Nacre<br>CS 30001<br>14033 CAEN cedex 9<br>Charlotte Gourio
500	J.H. de Vries<br>Academisch Medisch Centrum<br>Meibergdreef 9<br>1105 AZ Amsterdam	METC AMC<br>Meibergdreef 9<br>1105 AZ Amsterdam<br>Prof.dr. M.P.M. Burger
501	T. van Bemmel<br>Gelre Ziekenhuis Apeldoorn<br>Albert Schweitzerlaan 31<br>7334 DZ Apeldoorn	METC AMC<br>Meibergdreef 9<br>1105 AZ Amsterdam<br>Prof.dr. M.P.M. Burger
502	W.H. van Houtum<br>Spaarne Gasthuis<br>Spaarnepoort 1<br>2134 TM Hoofddorp	METC AMC<br>Meibergdreef 9<br>1105 AZ Amsterdam<br>Prof.dr. M.P.M. Burger

503	H.W. de Valk<br>Universitair Medisch Centrum Utrecht<br>Heidelberglaan 100<br>3584 CX Utrecht	METC AMC<br>Meibergdreef 9<br>1105 AZ Amsterdam<br>Prof.dr. M.P.M. Burger
504	E.J.P. de Koning<br>Leiden University Medical Center<br>Albinusdreef 2<br>2333 ZA Leiden	METC AMC<br>Meibergdreef 9<br>1105 AZ Amsterdam<br>Prof.dr. M.P.M. Burger
505	A.G. Lieverse<br>Maxima Medisch Centrum<br>Ds. Theodor Fliednerstraat 1<br>5631 BM Eindhoven	METC AMC<br>Meibergdreef 9<br>1105 AZ Amsterdam<br>Prof.dr. M.P.M. Burger
506	D. Mul<br>Diabeter<br>Blaak 6<br>3011 TA Rotterdam	METC AMC<br>Meibergdreef 9<br>1105 AZ Amsterdam<br>Prof.dr. M.P.M. Burger
508	C.J.J. Tack<br>Radboud UMC<br>G. Grooteplein-Zuid 8<br>6525 GA Nijmegen	METC AMC<br>Meibergdreef 9<br>1105 AZ Amsterdam<br>Prof.dr. M.P.M. Burger
509	A. Kooy<br>Bethesda Diabetes Research Center<br>Dr. G.H. Amshoffweg 1<br>7909 AA Hoogeveen	METC AMC<br>Meibergdreef 9<br>1105 AZ Amsterdam<br>Prof.dr. J.A. Swinkels
All RU sites: 601-612 (Central EC)	Ethics Committee at Ministry of Health of the Russian Federation<br>3, Rakhmanovskiy per., GSP-4,<br>127994 Moscow, Russia	Ethics Committee at Ministry of Health of the Russian Federation<br>3, Rakhmanovskiy per., GSP-4,<br>127994, Moscow, Russia<br>Prof. A. Chuchalin
601	Dr. Aleksandr Maiorov<br>Federal State Budgetary Institution ”Endocrinological Research Centre” of the Ministry of Healthcare of the Russian Federation<br>11, Dm. Ulyanova street 117036 Moscow	Ethics Committee of Federal State Budgetary Institution “Endocrinological Research Centre” of the Ministry of Healthcare of the Russian Federation<br>11, Dm. Ulyanova street 117036 Moscow<br>Prof. Rozhinskaya, L.Ya.
602	Dr. Lubov Kargina<br>State Healthcare Institution “Saratov City Clinical Hospital #12”<br>15, Krimskaya street 410039 Saratov	Ethics Committee of State Healthcare Institution “Saratov City Clinical Hospital #12”<br>15, Krimskaya street 410039 Saratov Ivanicheva, G.V.

603	Dr. Olga Khabarova<br>Budgetary Institution of the Chuvash Republic "Republican endocrinological dispensary" of the Ministry of Healthcare and Social Development of the Chuvash Republic<br>7, 139th Strelkovoi divizii street 428009 Cheboksary	Ethics Committee of the Federal State Educational Establishment of Higher Professional Education "Ulyanov, I. N. Chuvash State University"<br>45, Moscowsky Ave. 428023 Cheboksary<br>Chairperson: Prof. Volkov, V.E.<br><br>*Replaced with*<br><br>Ethics Committee of the Budgetary Institution of the Chuvash Republic "Republic Clinical Oncologic Dispensary"<br>23, F. Gladkov street 428020 Cheboksary<br>Chairperson: Gamanov, S.V.
605	Prof. Vladimir Potin<br>Federal State Budgetary Scientific Institution "D.O.Ott Research Institute of Obstetrics, Gynecology and Reproductology"<br>3, Mendeleyevskaya line 199034<br>Saint- Petersburg	Ethics Committee of Federal State Budgetary Scientific Institution "D.O.Ott Research Institute of Obstetrics, Gynecology and Reproductology"<br>3, Mendeleyevskaya line 199034 Saint-Petersburg<br>Prof. Potin, V.V.
606	Prof. NataliaVorokhobina Saint-Petersburg State Budgetary Healthcare Institution "City St. Elizabeth Hospital"<br>14, Vavilovih street 195257 Saint-Petersburg	Local Ethics Committee of<br>St. Elizabeth Hospital<br>14, Vavilovih street 195257 Saint-Petersburg<br>Vovk, A.V.
607	Dr. Lidia Belousova<br>"Astarta" LLC<br>5A, Galernyi proezd 199226 Saint-Petersburg	"Astarta", LLC Local Ethics Committee<br>5, Galernyi proezd 199226 Saint-Petersburg<br>Stroev, Yu.I.
608	Dr. Alsu Zalevskaya<br>Saint-Petersburg State Budgetary Healthcare Institution "City Multifield Hospital № 2"<br>5, Uchebnyi line 194354 Saint-Petersburg	Local Ethics Committee of Saint- Petersburg State Budgetary Healthcare Institution "City Multifield Hospital № 2"<br>5, Uchebnyi line 194354 Saint-Petersburg<br>Nesterko, A.O.
609	Prof. Vadim Klimontov<br>Federal State Budgetary Institution "Scientific Research Institute of Clinical and Experimental Lymphology"<br>2, Timakova street 630060 Novosibirsk	Ethics Committee of Federal State Budgetary Institution "Scientific Research Institute of Clinical and Experimental Lymphology" 2, Timakova street 630060 Novosibirsk<br>Letyagina, E.A.

610	Dr. Leylya Gaysina<br>Federal State Autonomous Educational Institution of the Higher Professional Education “Kazan (Privolzhsky) Federal University”<br>Legal address: 18, Kremlevskaya street 420008 Kazan<br>Actual address: 18, Volkova street 420012 Kazan	Local Ethics Committee of Federal State Autonomous Educational Institution of the Higher Professional Education “Kazan (Privolzhsky) Federal University”<br>74, Karl Marx street 420015 Kazan<br>Faizullin, R. I.
611	Dr. Elena Frolova<br>State Budgetary Institution of the Mari El Republic “Polyclinic No. 2 in Yoshkar-Ola”<br>56, Sovetskaya street 424004 Yoshkar-Ola	Ethics Committee of State Budgetary Institution of the Mari El Republic “Polyclinic No. 2 in Yoshkar-Ola” 56, Sovetskaya street 424004 Yoshkar-Ola<br>Kuznetsov, V.M.
612	Dr. Khavra Astamirova<br>Saint-Petersburg State Budgetary Healthcare Institution “City Policlinic No. 27”<br>Legal: 29, ‘A’ letter, Voznesensky Ave. 190068 Saint-Petersburg<br>Actual: 27, Voznesensky Ave. 190068 Saint-Petersburg	Local Ethics Committee of S.M. Kirov Military Medical Academy<br>6, Lebedev street 194044<br>Saint-Petersburg<br>Prof. Shustov, S.B.
700	Andrej Janez, MD, PhD<br>UNIVERSITY MEDICAL CENTRE LJUBLJANA<br>Department for endocrinology, diabetes and metabolic diseases<br>Zaloska cesta 7<br>SI- 1000 Ljubljana	The National Medical Ethics Committee<br>Stefanova 5<br>1000 Ljubljana, Slovenia<br>Bozidar Voljc, MD, PhD-chairperson
701	Milivoj Piletic, MD<br>GENERAL HOSPITAL NOVO MESTO<br>Diabetes outpatient clinic<br>Šmihelska cesta 1<br>SI- 8000 Novo mesto<br>Slovenia	The National Medical Ethics Committee<br>Stefanova 5<br>1000 Ljubljana, Slovenia<br>Bozidar Voljc, MD, PhD-chairperson
702	Prof. Tadej Battelino, MD, PhD<br>UNIVERSITY CHILDREN’S HOSPITAL<br>Department of endocrinology, diabetes and metabolic disease Bohoričeva 20<br>SI- 1000 Ljubljana<br>Slovenia	The National Medical Ethics Committee<br>Stefanova 5<br>1000 Ljubljana, Slovenia<br>Bozidar Voljc, MD, PhD-chairperson
N/A	*Not Applicable*	***(Main IEC)***<br>Dr Leslie Gelling<br>East of England – Cambridge South Research Ethics Committee<br>The Old Chapel<br>Royal Standard Place<br>Nottingham NG1 6FS

800	Dr Anna Brackenridge<br>NIHR Clinical Research Facility<br>Guy's Hospital<br>15th Floor, Tower Wing<br>Great Maze Pond<br>London SE1 9RT	**(Local IRB)**<br>HRA (on behalf of Guy's and St Thomas's NHS Foundation Trust)<br>The Old Chapel<br>Royal Standard Place<br>Nottingham<br>NG1 6FS
801	Dr Mark Evans<br>Wolfson Diabetes & Endocrine Clinic<br>Box 281<br>Addenbrookes Hospital<br>Cambridge<br>CB2 0QQ	**(Local IRB)**<br>HRA (on behalf of Cambridge University Hospital NHS Foundation Trust)<br>The Old Chapel<br>Royal Standard Place<br>Nottingham<br>NG1 6FS
802	Dr Niall Furlong<br>Diabetes Centre,<br>Marshalls Cross Road,<br>St Helens,<br>Merseyside WA9 3DA	**(Local IRB)**<br>HRA (on behalf of St Helen's and Knowsley Teaching Hospitals NHS Foundation Trust)<br>The Old Chapel<br>Royal Standard Place<br>Nottingham<br>NG1 6FS
803	Dr Sutapa Ray<br>Research Office<br>19 Wetherby Road<br>Harrogate District NHS Foundation Trust<br>Lancaster Park Road<br>Harrogate HG2 7SX	**(Local IRB)**<br>HRA (on behalf of Harrogate and District NHS Foundation Trust)<br>The Old Chapel<br>Royal Standard Place<br>Nottingham<br>NG1 6FS
804	Dr Lalantha Leelarathna<br>Manchester Diabetes Centre,<br>Manchester Royal Infirmary,<br>193 Hathersage Road,<br>Manchester<br>M13 0JE	**(Local IRB)**<br>HRA (on behalf of Central Manchester University NHS Foundation Trust)<br>The Old Chapel<br>Royal Standard Place<br>Nottingham<br>NG1 6FS
805	Prof David Russell-Jones<br>The Cedar Centre<br>Royal Surrey County Hospital<br>Egerton Road<br>Guildford<br>Surrey GU2 7XX	**(Local IRB)**<br>HRA (on behalf of Royal Surrey County Hospitals NHS Foundation Trust)<br>The Old Chapel<br>Royal Standard Place<br>Nottingham
901	Dr. Robert Jeffrey Silver MD<br>Southern New Hampshire Diabetes and Endocrinology<br>29 Northwest Boulevard<br>Nashua, NH 03063	Sterling Institutional Review Board<br>6300 Powers Ferry Road<br>Suite 600-351<br>Atlanta, GA 30339<br>Steven L. Saltzman, M.D.

902	Dr. Stephen L Aronoff MD<br>Research Institute Of Dallas<br>10260 North Central Expressway<br>Suite 100N<br>Dallas, TX 75231	Sterling Institutional Review Board<br>6300 Powers Ferry Road<br>Suite 600-351<br>Atlanta, GA 30339<br>Steven L. Saltzman, M.D.
903	Dr. William C Biggs MD<br>Amarillo Medical Specialists LLP<br>1215 South Coulter St.<br>Suite 400<br>Amarillo, TX 79106	Sterling Institutional Review Board<br>6300 Powers Ferry Road<br>Suite 600-351<br>Atlanta, GA 30339<br>Steven L. Saltzman, M.D.
904	Dr. Elizabeth Hackman Harris MD<br>University of North Carolina, UNC Diabetes Care Center<br>300 Meadowmont Village Circle<br>Suite 201<br>[illegible]	Office of Human Research Ethics 720 Martin Luther King Jr. Blvd Bldg #385, Second Floor CB# 7097 Chapel Hill, NC 27599<br>David Weber, MD, MPH, MPA
905	Dr. Carl D. Vance MD<br>Rocky Mountain Diabetes & Osteoporosis Ctr, PA<br>3910 Washington Parkway<br>Idaho Falls, ID 83404-7596	Sterling Institutional Review Board<br>6300 Powers Ferry Road<br>Suite 600-351<br>Atlanta, GA 30339<br>Steven L. Saltzman, M.D.
906	Dr. Anders Carlson MD<br>International Diabetes Center<br>3800 Park Nicollet Blvd.<br>Minneapolis, MN 55416	Park Nicollet IRB for HealthPartners Institute<br>3311 E. Old Shakopee Road<br>Bloomington, MN 55425<br>John Schousboe
907	Dr. Michael A. Dempsey MD<br>Endocrine And Metabolic Consultants<br>3200 Tower Oaks Blvd.<br>Suite 250<br>Rockville, MD 20852	Sterling Institutional Review Board<br>6300 Powers Ferry Road<br>Suite 600-351<br>Atlanta, GA 30339<br>Steven L. Saltzman, M.D.
908	Dr. Lyle C. Myers MD<br>Kentucky Diabetes-Endocrinology Center<br>1760 Nicholasville Road<br>Suite 502<br>Lexington, KY 40503	Sterling Institutional Review Board<br>6300 Powers Ferry Road<br>Suite 600-351<br>Atlanta, GA 30339<br>Steven L. Saltzman, M.D.
909	Dr. Mark P. Christiansen<br>Diablo Clinical Research, Inc.<br>2255 Ygnacio Valley Road<br>Suite M<br>Walnut Creek, CA 94598	Sterling Institutional Review Board<br>6300 Powers Ferry Road<br>Suite 600-351<br>Atlanta, GA 30339<br>Steven L. Saltzman, M.D.
910	Dr. Patricia L Bononi MD<br>Partners In Nephrology & Endocrinology<br>5171 Liberty Ave<br>Pittsburgh, PA 15224-2215	Sterling Institutional Review Board<br>6300 Powers Ferry Road<br>Suite 600-351<br>Atlanta, GA 30339<br>Steven L. Saltzman, M.D.

911	Dr. Gregg Gerety MD<br>Albany Medical College, Division of Community Endocrinology<br>1365 Washington Avenue<br>Suite 300<br>Albany, NY 12206	Western Institutional Review Board<br>1019 39th Avenue<br>Suite 120<br>Ouyallup, WA 98374<br>Donald A. Deieso, Ph.D
912	Dr. Prakash Seshadri MD<br>Christiana Care Health Services, Inc.<br>4735 Ogletown-Stanton Road<br>Suite 3203<br>Newark, DE 19713	Christiana Care Institutional Review Board<br>Helen F. Graham Cancer Center<br>4701 Ogletown-Stanton Road<br>Suite 2350<br>Newark, DE 19713<br>Steven Kushner, MD
913	Dr. Priscilla L. Hollander MD, PhD<br>Baylor Endocrine Center<br>3600 Gaston Ave<br>Wadley Tower, Suite 656<br>Dallas, TX 75246	Sterling Institutional Review Board<br>6300 Powers Ferry Road<br>Suite 600-351<br>Atlanta, GA 30339<br>Steven L. Saltzman, M.D.
914	Dr. Yshay Shlesinger MD<br>NorCal Endocrinology and Internal Medicine<br>111 Deerwood Road<br>Suite 180<br>San Ramon, CA 94583	Sterling Institutional Review Board<br>6300 Powers Ferry Road<br>Suite 600-351<br>Atlanta, GA 30339<br>Steven L. Saltzman, M.D.
915	Dr. David M. Huffman MD<br>University Diabetes & Endocrine Consultants<br>5616 Brainerd Road<br>Chattanooga, TN 37411	Sterling Institutional Review Board<br>6300 Powers Ferry Road<br>Suite 600-351<br>Atlanta, GA 30339<br>Steven L. Saltzman, M.D.
916	Dr. Paul C. Norwood Jr., MD<br>Valley Research<br>550 East Herndon Avenue<br>Suite 101<br>Fresno, CA 93720	Sterling Institutional Review Board<br>6300 Powers Ferry Road<br>Suite 600-351<br>Atlanta, GA 30339<br>Steven L. Saltzman, M.D.
917	Dr. Michael L. Reeves<br>Michael L. Reeves, M.D.<br>725 Glenwood Drive<br>Suite E688<br>Chattanooga, TN 37404-1192	Sterling Institutional Review Board<br>6300 Powers Ferry Road<br>Suite 600-351<br>Atlanta, GA 30339<br>Steven L. Saltzman, M.D.
918	Dr. David M. Kayne MD<br>Medical Group Of Encino<br>16030 Ventura Blvd.<br>Suite 680<br>Encino, CA 91436	Sterling Institutional Review Board<br>6300 Powers Ferry Road<br>Suite 600-351<br>Atlanta, GA 30339<br>Steven L. Saltzman, M.D.

919	Dr. Tira Chaicha-Brom MD<br>Texas Diabetes & Endocrinology, P.A.<br>5000 Davis Lane<br>Suite 200<br>Austin, TX 78749	Sterling Institutional Review Board<br>6300 Powers Ferry Road<br>Suite 600-351<br>Atlanta, GA 30339<br>Steven L. Saltzman, M.D.
920	Dr. Elliot Eisenbud MD<br>NorCal Endocrinology and Internal Medicine<br>729 Sunrise Ave<br>Suite 501<br>Roseville, CA 95661	Sterling Institutional Review Board<br>6300 Powers Ferry Road<br>Suite 600-351<br>Atlanta, GA 30339<br>Steven L. Saltzman, M.D.
921	Dr. Luis Fernando Soruco MD<br>Northwest Endo Diabetes Research, LLC<br>2101 S Arlington Heights Rd<br>Suite 111<br>Arlington Heights, IL 60005-4144	Sterling Institutional Review Board<br>6300 Powers Ferry Road<br>Suite 600-351<br>Atlanta, GA 30339<br>Steven L. Saltzman, M.D.
922	Dr. Sumana Gangi MD<br>Southern Endocrinology Associates PA<br>1621 North Belt Line Road<br>Suite A<br>Mesquite, TX 75149	Sterling Institutional Review Board<br>6300 Powers Ferry Road<br>Suite 600-351<br>Atlanta, GA 30339<br>Steven L. Saltzman, M.D.
923	Dr. Larry D. Stonesifer MD<br>Larry Stonesifer, MD Inc., PS<br>34509 9th Avenue South<br>Suite 200 & 203A<br>Federal Way, WA 98003	Sterling Institutional Review Board<br>6300 Powers Ferry Road<br>Suite 600-351<br>Atlanta, GA 30339<br>Steven L. Saltzman, M.D.
924	Dr. David C. Klonoff MD<br>Mills-Peninsula Health Services, Diabetes Research Institute<br>100 South San Mateo Drive<br>5th Floor<br>San Mateo, CA 94401	Sutter Health Institutional Review Board<br>2121 N. California Blvd, Suite 310<br>Walnut Creek, CA 94596<br>David Gill, PhD, CIP
925	Dr. Ronald L. Brazg MD, FACE<br>Rainier Clinical Research Center Inc<br>723 Southwest 10th Street<br>Suite 100<br>Renton, WA 98057	Sterling Institutional Review Board<br>6300 Powers Ferry Road<br>Suite 600-351<br>Atlanta, GA 30339<br>Steven L. Saltzman, M.D.
926	Dr. Wendy S. Lane MD<br>Mountain Diabetes & Endocrine Center<br>1998 Hendersonville Highway Bldg 31<br>Asheville, NC 28803	Sterling Institutional Review Board<br>6300 Powers Ferry Road<br>Suite 600-351<br>Atlanta, GA 30339<br>Steven L. Saltzman, M.D.
927	Dr. Kristin Castorino DO William Sansum Diabetes Center 2219<br>Bath Street<br>Santa Barbara, CA 93105	Sterling Institutional Review Board<br>6300 Powers Ferry Road<br>Suite 600-351<br>Atlanta, GA 30339<br>Steven L. Saltzman, M.D.

928	Dr. Samer N. Nakhle MD<br>Palm Research Center, Inc.<br>9280 West Sunset Road<br>Suite 306<br>Las Vegas, NV 89148	Sterling Institutional Review Board<br>6300 Powers Ferry Road<br>Suite 600-351<br>Atlanta, GA 30339<br>Steven L. Saltzman, M.D.
929	Dr. Elena Toschi<br>Joslin Center For Diabetes<br>1 Joslin Place<br>Boston, MA 02215	Joslin Diabetes Center<br>Committee on Human Services<br>One Joslin Place<br>Boston, MA 02215<br>Robert C. Stanton, M.D
930	Dr. Bruce W Bode MD<br>Atlanta Diabetes Associate<br>1800 Howell Mill Road<br>Suite 450<br>Atlanta, GA 30318	Sterling Institutional Review Board<br>6300 Powers Ferry Road<br>Suite 600-351<br>Atlanta, GA 30339<br>Steven L. Saltzman, M.D.
